# Down-regulation of Sox7 is associated with aberrant activation of Wnt/β-catenin signaling in endometrial cancer

**DOI:** 10.18632/oncotarget.667

**Published:** 2012-11-07

**Authors:** David W Chan, Celia SL Mak, Thomas HY Leung, Karen KL Chan, Hextan YS Ngan

**Affiliations:** ^1^ Departments of Obstetrics and Gynaecology, LKS Faculty of Medicine, the University of Hong Kong, Hong Kong SAR, P.R.China

**Keywords:** Sox7, Wnt/β-catenin, endometrial cancer, TCF/LEF-1, CyclinD1, FGF9

## Abstract

Although the mortality rate of endometrial cancer is comparatively low in gynecologic malignancies, a rising trend of this cancer has been observed for the past decade. The understanding of the molecular mechanism will favor for the clinical management of this disease. Aberrant activation of Wnt/β-catenin signaling pathway plays a major role in the pathogenesis of endometrioid adenocarcinoma including this cancer type. In this study, we reported that Sox7, one of Sox transcriptional factors, was frequently underexpressed in endometrial cancer and importantly, it was associated with dysregulation of the Wnt/β-catenin signaling activity. Immunohistochemical and quantitative RT-PCR analyses showed that Sox7 was underexpressed and was associated with high-grade tumor (*P*=0.021), increased expressions of β-catenin (*P*=0.038) and its downstream targets; CyclinD1 (*P*<0.001) and FGF9 (*P*<0.001). In addition, using HEK293T cell model, we found that Sox7 was able to inhibit TCF/LEF-1-dependent luciferase activity induced by Wnt-1. This was further proved by that Sox7 could significantly suppress the expressions of Wnt targets; Cyclin D1 and C-myc in endometrial cells. Immuno-fluorescent microscopy revealed that Sox7 was co-localizaed with either mutant β-catenin or TCF4 protein in nucleus, while co-immunopreciptation assay demonstrated that Sox7 could physically interact with not only wild-type but also mutant β-catenin, as well as TCF4 proteins. Functionally, enforced expression of Sox7 could significantly inhibit endometrial or endometrioid ovarian cancer cells (OEA) harboring either wild-type or mutant β-catenin. These data suggest Sox7 is a negative regulator of Wnt/β-catenin signaling pathway through impeding the transcriptional machinery of β-catenin/TCF/LEF-1 transcriptional complex, and the loss of expression may be involved in the pathogenesis of endometrial cancer.

## INTRODUCTION

Endometrial carcinoma is one of the common female malignancies in the western countries but it is getting common in Asian including Hong Kong Chinese. According to the Hong Kong Cancer Registry from 1999 to 2007, endometrial carcinoma has increased from the ninth to the fourth most common gynecologic malignancies for females overall in Hong Kong (Hong Kong Cancer Registry 2007). Although the mortality rate of this disease is keeping at approximately 1.0%, a significant increase for the incidence trend has been revealed from 1999 to 2007 (Hong Kong Cancer Registry 2007). This indicates endometrial cancer becomes more common in Hong Kong women nowadays. There are two primary types of endometrial cancer according to histopathology, cell biology and clinical course [1]. Endometrioid adenomacarcinoma or Type I carcinoma are the most common type and estrogen-related, often preceded by hyperplasia. Type II or non-endometrioid endometrial cancer usually exhibit serous or clear cell differentiation and estrogen-independent.

Wnt/β-catenin or canonical Wnt signal transduction pathway is a conserved molecular mechanism in metazoan animals. This signaling pathway regulates a remarkable variety of cellular process such as cell fate, cell proliferation, cell survival, cell behavior and migration [2]. In this pathway, β-catenin is the most effector for the regulation of Wnt/β-catenin targets. The stability of β-catenin is regulated by a multiprotein complex such as glycogen synthase kinase-3β (GSK3β) and casein kinase 1 (CK1), and the scaffolding proteins adenomatous polyposis coli (APC), Axin1 and Axin2 (conductin). Aberrant activation of this pathway due to the accumulation of β-catenin is commonly found in human cancers [3-6]. Mounting evidences have revealed that the oncogenic mutations in *β-catenin*, *APC* and *Axins* are often associated with the upregulation of β-catenin and the pathogenesis of endometrioid-type of endometrial cancer and ovarian cancer [7-10]. The accumulated β-catenin eventually translocates into nucleus promotes tumor progression through its persistent interaction with one or more of its numerous downstream targets such as TCF/LEF factor. Therefore, promoting β-catenin degradation by targeting the upstream sites of Wnt/β-catenin pathway is a rational approach for the therapy of cancers [11, 12]. However, most of human cancers including endometrial cancer (38%) harbor mutations on serine/threonine residues (codons 33, 37, 41, and 45) of β-catenin [13-15]. This causes difficulties of using above ways to inhibit the aberrant activation of Wnt/β-catenin signaling pathway in this disease.

In this study, we identified Sox7, one of the Sox transcription factors family, was significantly down-regulated in high-grade endometrial cancer and inversely correlated with Wnt/β-catenin signaling activity. Enforced expression of Sox7 could remarkably suppress not only Wnt/β-catenin signaling and its downstream oncogenes, but also endometrial cancer cell growth. Importantly, we demonstrated that Sox7 inhibited Wnt/β-catenin signaling in endometrial or endometrioid ovarian cancer cells (OEA) harboring not only wild-type but also mutant β-catenin. Our findings manifest the significance of Sox7 in the pathogenesis and the regulatory mechanisms of aberrant activation of Wnt/β-catenin signaling activity in endometrial cancer. This new knowledge generated may reveal novel strategies for rational design of Wnt/β-catenin inhibiting agents to combat this cancer.

## MATERIALS AND METHODS

### Clinical samples and cell lines

Surgical resection of 43 tumor samples from primary endometrial cancer patients and 37 normal endometrial samples from benign diseases such as uterine fibroid after surgery were randomly chosen for Q-PCR analysis. The histology of all endometrial cancer tissue samples have been verified by surgical pathologists. The histological subtype and stage of the tumors were categorized according to International Federation of Gynaecology and Obstetrics (FIGO) classification. Written informed consent was taken and the use of these clinical samples was approved by Institutional Review Board of the University of Hong Kong/ Hospital Authority Hong Kong West Cluster (HKU/HA HKW IRB)(Institutional Review Board number: UW08-069). Five endometrial cancer cell lines (RL95, KLE, HEC-1A and HEC-1B) (America Type Culture Collection, Rockville, MD, USA) and Ishikawa (ECACC 99040201, Sigma, St. Louis, MO) were used for investigation of Sox7 functions. Human Embryonic Kidney 293 cells (HEK 293) (ATCC) was used for TOP/FOP luciferase reporter assay, and L Wnt3A cell line (CRL-2647) was used for Wnt3A conditioned medium (ATCC). The cell lines authentication was done by in-house STR DNA profiling analysis and were cultured at 37°C in 5% CO_2_ in Dulbecco's modified Eagle medium (DMEM/F12) (Gibco-BRL, Gaithersburg) with 10% fetal bovine serum (Gibco) and 1% Penicillin-Streptomycin (Gibco).

### Plasmids and cell transfection

The Flag/Sox7 expressing plasmid (gift from Dr. Hayashi Y, Department of Genetics, Nagoya University, Japan) was for ectopic expression of Flag-tagged Sox7 fusion protein. The Sox7 cDNA was subcloned into pEGFP-C1 (Clontech, Mountain View, CA, USA) to generate GFP/Sox7 expressing plasmid. The HA/β-catenin expressing plasmid (gift from Dr. Muller AG, Max-Planck-Institut, Germany) and the GFP/mutant β-catenin S37A expressing plasmid (gift from Dr. Wong AS, School of Biological Sciences, The University of Hong Kong, Hong Kong) were used for expressing wild-type and mutant β-catenin respectively. The Myc/TCF4 expressing plasmid (gift from Dr. Idogawa M, cancer Research Institute, Sapporo Medical University, Japan) was used for expressing Myc/TCF4 fusion protein. The pSuper8XTOPFlash and pSuper8XFOPFlash plasmids (gift from Dr. Moon R, University of Washington, USA) were used for TOP/FOP luciferase reporter assay. The pLNCX-Wnt1 construct (kindly provided by Dr. M. Semenov, Harvard University, USA) was used for induction of Wnt/β-catenin activity. Lipofectamine™ LTX was used for cell transfection according to the manufacturer's instructions (Invitrogen Life Technologies, Carlsbad, CA, USA).

### RNA extraction and real time quantitative RT-PCR

The total RNA was isolated from clinical samples and cell lines by TRIzol reagent (Invitrogen). The cDNA was synthesized using Reverse transcription reagent kit (Applied Biosystems, Foster City, CA, USA). Real time quantitative reverse transcriptase-PCR (Q-PCR) was used for evaluating the expressions of *Sox7*, *FGF9*, *SFN*, *Cyclin D1* and *C-myc* using Taqman® Gene expression Assays; human *Sox7* (Assay ID: Hs00846731_s1), human *FGF9* (Assay ID: Hs00181829_m1), human *SFN* (Assay ID: Hs00968567_s1), human *Cyclin-D1* (Assay ID: Hs00765553_m1) and human *C-myc* (Assay ID: Hs00153408_m1), in an ABI PRISM™ 7500 system (Applied Biosystems). The human *18S rRNA* (Assay ID: Hs99999901_m1) was used as an internal control.

### Western blot and co-immunoprecipitation assays

Cell lysate was prepared from cells using lysis buffer (Cell Signaling Technology, Darvers, MA, USA) containing protease inhibitor (Sigma) and Phenylmethylsulfonyl fluoride (PMSF) (Sigma Chemical Co., St Louis, MO, USA). The equal amount of protein samples was separated by 10% SDS-PAGE and electroblotted onto the Hybond-P membranes (Amersham Pharmacia Biotech, Cleveland, OH, USA). Blots were blocked with 5% skim milk, followed by incubation with β-catenin (Cell Signaling) and C-Myc (N262) (Santa Cruz Biotechnology, Inc., Santa Cruz, CA, USA), Cyclin-D1 (Cell Signaling), anti-HA (Roche Applied Science, Indianapolis, IN, USA), anti-GFP (Santa Cruz), anti-Flag and β-actin (Sigma) overnight at 4°C. Blots were then incubated with anti-mouse or anti-rabbit (Amersham Pharmacia Biotechnology) secondary antibodies conjugate with horseradish peroxidase for 1 hour in room temperature and visualized using ECL™ Western Blotting Detection Reagent (Amersham).

For co-immunoprecipitation assay, HEK293 cells were transiently co-transfected with Flag/Sox7 and HA/β-catenin, or GFP/mutant β-catenin, or Myc/TCF4 plasmids. The procedure of immunoprecipitation was performed as previously described [16, 17].

### Immunohitochemical and Immunofluorescent analyses

For immunohistochemical analysis, a commercial ovarian cancer tissue array (EMC1501, Pantomics Inc, San Francisco, CA) was immunostained with primary rabbit polyclonal anti-Sox7 (R&D Systems, Minneapolis, MN USA) in 1:250 dilution, anti-β-catenin (BD Biosciences, St Jose, CA, USA) in 1:400 dilution, and anti-FGF9 (Santa Cruz) in 1:200 dilution. The immunoreactivity of immuno-positive sample was determined by multiplying the intensity of the staining (+1, faint, +2 moderate, +3 strong and +4 very strong) and percentage of stained area (0-100%). The mean of immunoreactivity value of normal and borderline cases was used to normalize all cases. The examination and scoring of all tissues were done by two investigators independently.

For immunofluorescent analysis, HEC1B cells were cultured on cover slips and transiently transfected with either RFP/Sox7 or GFP/mutant β-catenin S37A, or GFP/Sox7 and Flag/TCF4 and Myc/TCF4 expressing plasmids. After 24 hours, the transfected cells were fixed with 4% paraformaldehyde, treated with 0.1% Triton, incubated with anti-Flag (Sigma) and Alexa Fluor® Fluorescent Streptavidin Conjugates (Invitrogen), followed by counter-stained with DAPI (4',6-diamidino-2-phenylindole) (Invitrogen), the fluorescent signals were examined and photographed by fluorescent microscopy (Leica Q550CW).

### Luciferase Reporter Assay

HEK293 cells were seeded in 24-well plates and transiently transfected with various amounts (0, 250 and 500 ng) of Flag/Sox7, 100 ng pLNCX-Wnt1, a pSuper8XTOPFlash or pSuper8XFOPFlash luciferase reporter constructs. All transfections were normalized with pcDNA vector and incubated for 24 hours. The luciferase activity was measured using the Dual-luciferase Reporter Assay System (Promega) and transfection efficiency was normalized with *Renilla* luciferase activity. All experiments were repeated in 3 independent experiments.

### Cell viability analysis

Cell viability was evaluated by Cell Proliferation Kit II (XTT) for 5 days according to the manufacturer's instructions (Roche). The experiment was repeated in three independent experiments.

### Statistical analysis

Student's *t* test (for parametric data) and the Mann-Whitney test (for non-parametric data) were used. Statistical analyses on clinicopathological correlation were performed using the SPSS version 13.0 software (SPSS, Chicago, IL, USA). A *p*-value was considered significant when less than 0.05.

## RESULTS

### Sox7 is frequently down-regulated and is associated with high Wnt/β-catenin signaling activity in endometrial cancer

**Figure 1:**
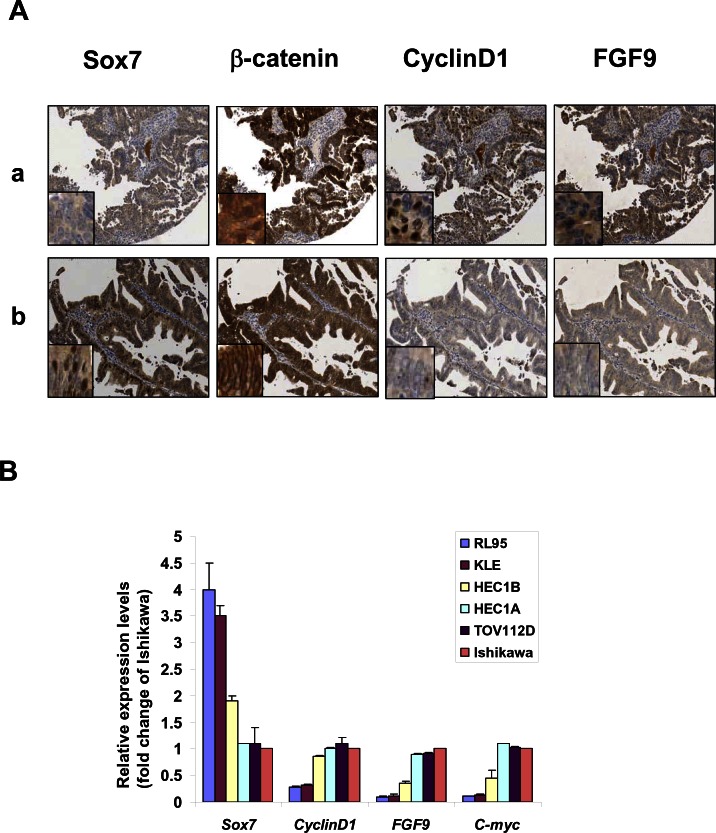
Sox7 is frequently underexpressed and inversely correlated with Wnt/β-catenin signaling in endometrial cancer (A) Immunohistochemical analysis showed the presence of Sox7 could significantly reduce the expressions of CyclinD1 and FGF9 in β-catenin overexpressing endometrial cancer (x20). Nuclear Sox7, CyclinD1 and FGF9 immunoreactivities were commonly found in endometrial cancer. The immunoreactivity of β-catenin could be found in the nucleus, cytoplasm and membrane. (B) Real time quantitative RT-PCR analysis showed that underexpressed *Sox7* was correlated with the increased expressions of *CyclinD1*, *FGF9* and *C-myc* in Wnt/β-catenin signaling active endometrial cancer cell lines (HEC1B, HEC1A and Ishikawa) and an OEA cell line (TOV112D).

Emerging reports have documented that Sox7 is down-regulated in several human cancers and may be a negative regulator of Wnt/β-catenin signaling activity [18-20]. As aberrant activation of Wnt/β-catenin signaling [8, 21], we thus firstly evaluated the expression status of Sox7 in endometrial carcinoma. By real-time quantitative RT-PCR (Q-PCR) analysis, we found that the expression levels of *Sox7* in endometrial cancer was 4.2-fold less than normal endometrium (*P* = 0.005) (see Supplementary Fig. 1). To investigate the association of *Sox7* expression with Wnt/β-catenin signaling activity in endometrial carcinoma, two common Wnt/β-catenin specific targets in endometrioid type cancers; Fibroblast growth factor 9 (*FGF9*) and Stratifin (*SFN*) [22, 23] were selected for Q-PCR analysis. Our finding showed that there was a reciprocal relationship between Sox7 and the levels of *FGF9* and *SFN* which were 17.9-fold (*P* = 0.048) and 7.27-fold (*P* = 0.008), respectively, higher in endometrial cancer as compared with normal endometrium (see Supplementary Fig. 1). To further investigate the expression status of Sox7 and Wnt/β-catenin activity, we evaluated the expressions of Sox7, β-catenin, CyclinD1 and FGF9 by immunohistochemical analysis on a human endometrial cancer array (EMC1501, Pantomics, Inc). Positive staining for Sox7 expression was detected in 100% (5/5) of normal and atypical hyperplasia, while 42.5% (51/120) of endometrioid endometrial cancer samples showed relatively lower or undetectable levels of Sox7 (Table 1). Moreover, we observed that Sox7 expressed mainly in nucleus and partially in cytoplasm of endometrial cancer cells (Fig. 1A). Clinicopathological correlation revealed that the down-regulated Sox7 was significantly associated with high-grade tumor (*P* = 0.021) and high level of β-catenin (*P* = 0.038). On the other hand, we found that 51.7% (62/120) endometrial cancer cases showed relatively higher in β-catenin expression (>2.7 folds) (Table 2), but only 20% (1/5) of normal and atypical hyperplasia cases showed higher β-catenin in cytoplasm and at membrane. In contrast to Sox7, the upregulated β-catenin was correlated with high-grade tumor (*P* = 0.045), and two Wnt/β-catenin specific downstream targets; Cyclin D1 (*P* < 0.001) and FGF9 (*P* = 0.003) [23, 24]. This indicates that there is a trend of inverse expression pattern between Sox7 and β-catenin in endometrial cancer.

**Table 1:** Clinicopathological correlation of Sox7 expression in endometrial cancer tissue array (EMC1501) (Pantomics, CA, USA)

Characteristics	Total	Sox7 expression (fold change)		
		< 2.2 folds	> 2.2 folds	p
All cases	120	51 (42.5%)	69 (57.5%)	
Stage				
Early (1)	84	55 (65.5%)	29 (34.5%)	
Late (2+3)	34	20 (58.8%)	14 (41.2%)	0.531
Grade				
Low (1+2)	66	38 (57.6%)	28 (42.4%)	
High (3)	40	32 (80.0%)	8 (20.0%)	0.021*
Lymph Node Metastasis				
Absence	95	60 (63.2%)	35 (36.8%)	
Presence	23	15 (65.2%)	8 (34.8%)	1.000
β-catenin				
<2.7 folds	58	42 (72.4%)	16 (27.6%)	
>2.7 folds	62	33 (53.2%)	29 (46.8%)	0.038*
CyclinD1				
<2.5 folds	56	45 (80.4%)	11 (19.6%)	
>2.5 folds	64	30 (46.9%)	34 (53.1%)	<0.001*
FGF9				
<3.0 folds	51	43 (84.3%)	8 (15.7%)	
>3.0 folds	69	32 (46.4%)	37 (53.6%)	<0.001*

**Table 2:** Clinicopathological correlation of β-catenin expression in endometrial cancer tissue array (EMC1501) (Pantomics, CA, USA)

Characteristics	Total	β-catenin expression (fold change)		
		< 2.7 folds	> 2.7 folds	p
All cases	120	58 (48.3%)	62 (51.7%)	
Stage				
Early (1)	84	44 (52.3%)	40 (47.7%)	
Late (2+3)	34	14 (41.2%)	20 (58.8%)	0.313
Grade				
Low (1+2)	66	37 (56.1%)	29 (43.9%)	
High (3)	40	14 (35.0%)	26 (65.0%)	0.045*
Lymph Node Metastasis				
Absence	95	50 (52.6%)	45 (47.4%)	
Presence	23	8 (34.8%)	15 (65.2%)	0.164
CyclinD1				
<2.5 folds	56	38 (67.9%)	18 (32.1%)	
>2.5 folds	64	20 (31.3%)	44 (68.7%)	<0.001*
FGF9				
<3.0 folds	51	33 (64.7%)	18 (35.3%)	
>3.0 folds	69	25 (36.2%)	44 (63.8%)	0.003*

To investigate the suppression effects of Sox7 on Wnt/β-catenin activity of endometrial cancer, we particularly examined the expression levels of CyclinD1 and FGF9 in β-catenin highly expressing endometrial cancer samples which had either with or without expression of Sox7. IHC analysis displayed that both CyclinD1 and FGF9 were expressed as highly as β-catenin in Sox7 underexpressed sample (Fig. 1A). In contrast, both CyclinD1 and FGF9 were remarkably reduced in Sox7 expressing sample (Fig. 1A). We also evaluated the expression of *Sox7* in human endometrial cancer cell lines by Q-PCR analysis. Of 5 endometrial cell lines (RL95, KLE, HEC-1B, HEC-1A, Ishikawa and an ovarian endometrioid carcinoma cell line (OEA) (TOV112D)), HEC-1A, HEC-1B, Ishikawa and TOV112D which have previously showed high Wnt/β-catenin signaling activity [22, 25] expressed lower *Sox7* but higher levels of *CyclinD1*, *C-myc* and *FGF9* (Fig. 1B) Taken together, these findings suggest that Sox7 may be a negative regulator of Wnt/β-catenin signaling activity and is frequently down-regulated in endometrial cancer.

### Sox7 is a negative regulator of Wnt/β-catenin signaling

**Figure 2:**
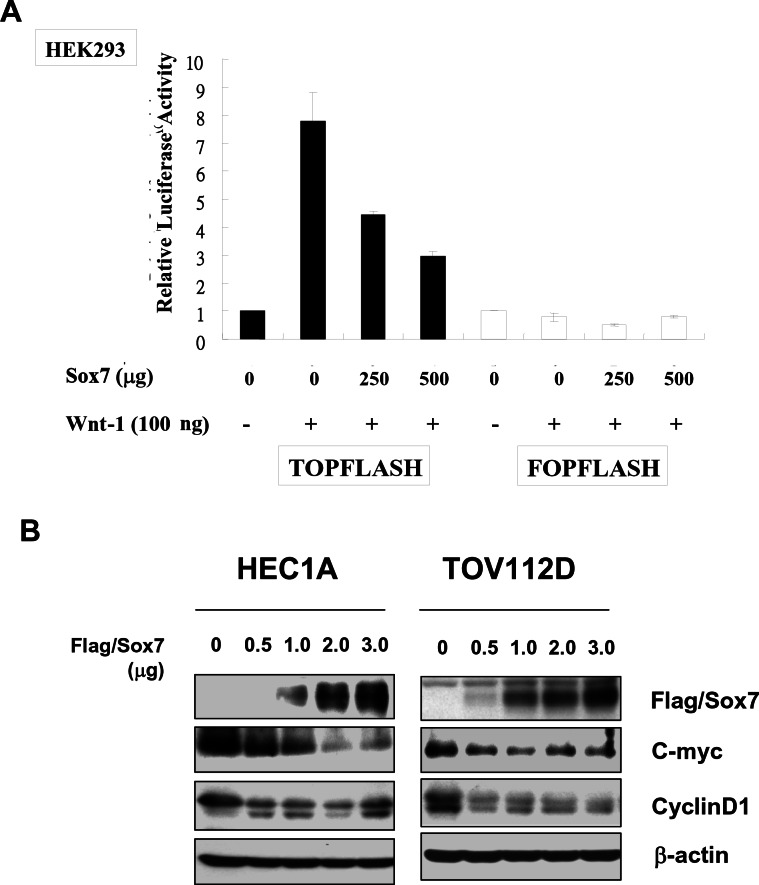
Sox7 is able to negatively regulate Wnt/β-catenin signaling activity (A) Sox7 could reduce Wnt-1 induce TOPFLASH luciferase activity dose dependently in HEK293 cells. FOPFLASH luciferase activity was used as a negative control. (B) Transient transfection of Flag/Sox could remarkably reduce the expressions of C-myc and cyclinD1 in HEC1A (wild-type β-catenin) and TOV112D (mutant β-catenin) cells.

To further demonstrate that Sox7 is able to suppress Wnt/β-catenin activity, we performed TOPFlash reporter assay using HEK293 cells [16]. Transfection of Wnt-1 expressing construct into HEK293 cells resulted in a 7.8-fold increase in β-catenin transcriptional activity. When Sox7 was co-transfected with Wnt-1, the β-catenin transcriptional activity was inhibited in a dose-dependent manner of Sox7 (*P* < 0.01) (Fig. 2A). Moreover, stably ectopic expression of Sox7 could remarkably reduce two Wnt/β-catenin downstream targets; C-myc and CyclinD1, in two Sox-7 deficient cell lines; HEC-1A and TOV112D, in a dose-dependent manner (Fig. 2B). Intriguingly, this inhibitory effect of Sox7 on Wnt/β-catenin signaling was not only restricted in wild-type β-catenin endometrial cancer cell line (HEC-1A) but also in mutant β-catenin OEA cells (TOV-112D) (Fig. 2B). Taken together, these data further implicate that Sox7 has inhibitory effect on Wnt/β-catenin activity in endometrial cancer cells harboring not only wild-type but also mutant β-catenin.

### Sox7 impedes Wnt/β-catenin signaling mediated endometrial cancer cell growth

**Figure 3:**
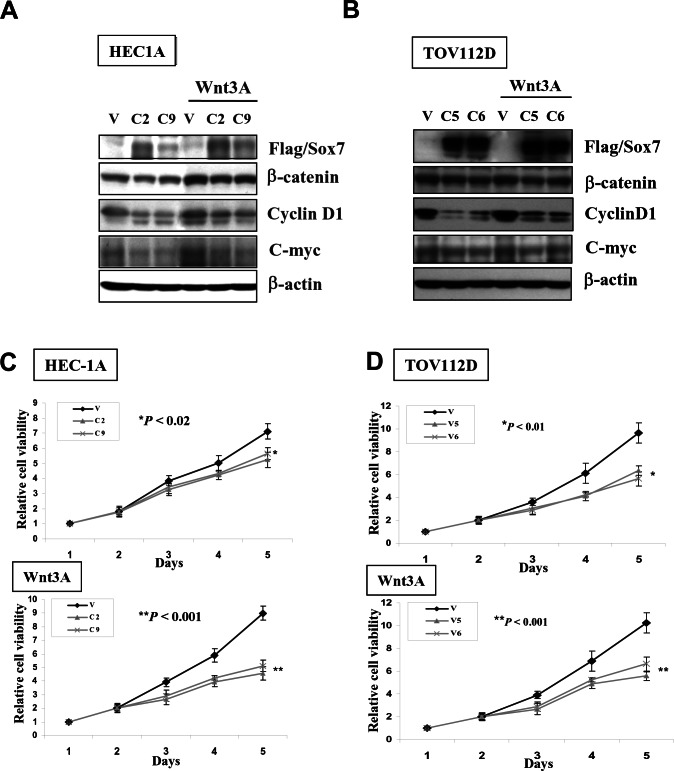
Enforced expression of Sox7 inhibits not only Wnt/β-catenin signaling activity but also cell growth of endometrial cancer cells (A) Western blotting showed Sox7 could suppress the expressions of C-myc and CyclinD1 in Sox7 stable expression clones in HEC1A and TOV112D cultured in normal and Wnt3A condition media. (B) XTT cell proliferation assay demonstrated that Sox7 could significantly inhibit cell growth of Sox7 stable expression clones in HEC1A and TOV112D cultured in normal and Wnt3A condition media.

As the aberrant activation of Wnt/β-catenin signaling is involved in promoting cancer cell growth, it is of interest to examine the suppressive role of Sox7 in the cell growth capacity of endometrial cancer. We firstly generated stable expressing Sox7 clones in a wild-type β-catenin cell line HEC-1A (C2 and C9), and a mutant β-catenin cell line TOV112D (C5 and C6). Western blotting showed that the ectopic expression of Sox7 was accompanied with the reduced expression of CyclinD1 and C-myc in all clones of both cell lines (Fig. 3A & 3B). When co-treatment of Wnt3A conditioned medium, HEC-1A with wild-type β-catenin was markedly elevated in β-catenin, CyclinD1 and C-myc expressions. But the enforced expression of Sox7 could inhibit both CyclinD1 and C-myc significantly (Fig. 3A). On the other hand, the level of mutant β-catenin in TOV112D could not further be elevated upon treatment of Wnt3A, while Sox7 still had a suppressive effect on the expressions of CyclinD1 and C-myc (Fig. 3B). With XTT cell proliferation assay, we found that the Sox7 stably expressing clones of HEC-1A (C2 and C9) (*P* < 0.02) and TOV112D (C5 and C6) (*P* < 0.01) exhibited significant reduction of cell proliferation rate (28 to 35%) as compared with their vector controls (Fig. 3C & 3D). Upon treatment of Wnt3A conditioned medium, all Sox7 stable expressing clones in HEC-1A (*P* < 0.001) and TOV112D (*P* < 0.001) further displayed suppressive effect on cell proliferation rate as compared with their vector controls (Fig. 3C & 3D). These data suggest that Sox7 exerts inhibitory effect on Wnt/β-catenin signalling mediated cell growth in endometrial cancer cells.

### Sox7 interacts with wild-type and mutant β-catenin, as well as TCF4

**Figure 4:**
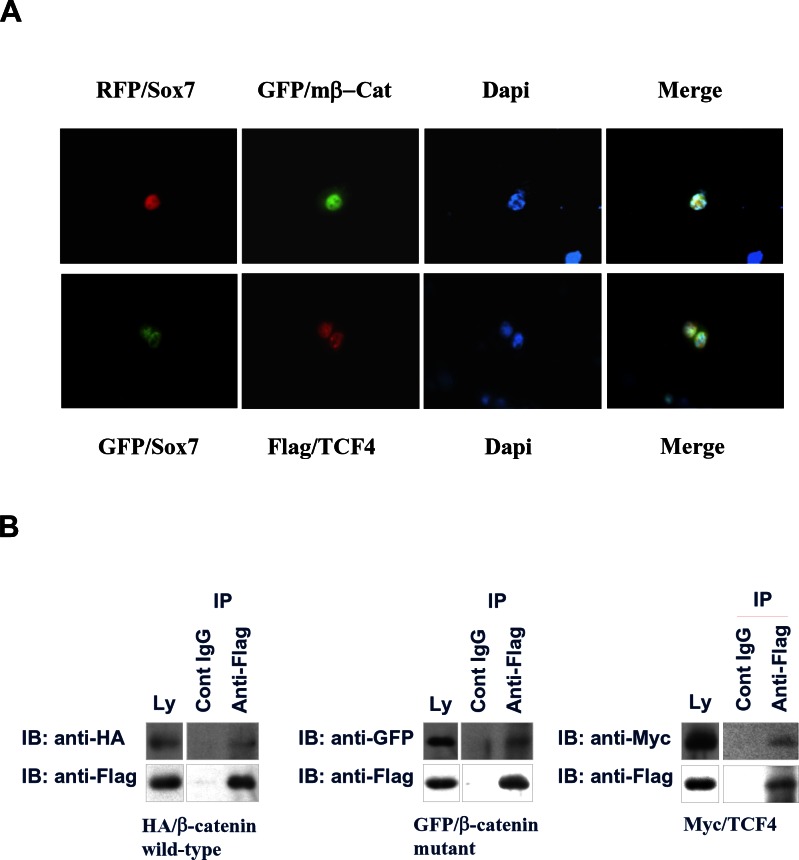
Sox7 interacts with β-catenin and TCF4 in the nucleus of endometrial cancer cells (A) Immunofluorescent analysis showed that Sox7 (RFP-Sox7 (red) and GRF-Sox7(green)) co-localized with mutant β-catenin (GFP-mβ-Cat)(green) and Flag/TCF4 (red) in the nucleus of HEC1A cells. (B) Co-immunoprecipitation assay showed that Sox7 (Flag/Sox7) could interact with wild-type β-catenin (HA/β-catenin), mutant β-catenin (GFP/β-catenin mutant), and TCF4 (Myc/TCF4). IgG was used as a control antibody. Ly, total lysate control.

Given that Sox7 can negatively regulate Wnt/β-catenin signaling activity through reduction of its downstream targets, it may interrupt the transcriptional activity of this pathway. Therefore, we were interested in whether Sox7 would interact with transcriptional factor complex β-catenin/TCF/LEF. We firstly examined the localization of Sox7, β-catenin and TCF4 in endometrial cancer cells, HEC-1A. By immunofluorescent microscopy, we demonstrated that RFP/Sox7 or GFP/Sox7 was localized mainly in the nucleus (Fig. 4A). Similarly, both GFP/mutant β-catenin S37A and Flag/TCF4 were also localized in the nucleus (Fig. 4A), indicating that all of these factors were co-localized in the nucleus of endometrial cancer cells. On the other hand, by co-immunoprecipitation assay, the Flag/Sox7 could strongly interact with HA/β-catenin, GFP/mutant β-catenin S37A, as well as Myc/TCF4 (Fig. 4B). These data indicate that Sox7 could interact with multiple factors such as wild-type and mutant β−catenin, as well as TCF4 in the nucleus of endometrial cancer cells. The interaction of Sox7 with these factors may impair the transcription activity of β-catenin/TCF/LEF transcriptional factor complex.

## DISCUSSION

This study showed that Sox7 was a potent negative regulator in the Wnt/β-catenin signaling pathway and was frequently underexpressed in endometrial cancer. The underexpressed Sox7 was associated with increased Wnt/β-catenin signaling activity and high-grade endometrial cancer. Importantly, we demonstrated that the enforced expression of Sox7 remarkably impeded Wnt/β-catenin signaling mediated cancer cell growth and such suppressive effect was independent on the presence of wild-type or mutant β-catenin in endometrial cancer or OEA cells.

Previous studies documented that approximately 40% of endometrial cancer have aberrant activation of Wnt/β-catenin signaling pathway [7, 8]. Most of the cases are due to oncogenic mutations in the β-catenin, APC and Axins [7, 8]. Interestingly, many studies have demonstrated that the endometrioid-type of endometrial cancer and ovarian cancer have similar Wnt/β-catenin abnormalities [9, 10]. Our findings from IHC analysis showed that β-catenin was highly expressed in the nuclei, cytoplasm and membrane of endometrial cancer samples. Clinic-pathological analysis revealed that the upregulated β-catenin was correlated with high-grade tumor. Intriguingly, Sox7 was commonly down-regulated in endometrial cancer samples. This finding is in agreement with previous reports on that the dysregulation of Wnt/β-catenin signaling pathway is linked to the advanced-stage of human colorectal, lung and prostate cancers [19, 20, 26]. On the other hand, the underexpressed Sox7, in contrast to β-catenin, was also associated with high-grade tumor. This indicates that the expressions of Sox7 and β-catenin have an inverse relationship. Our IHC result showed that Sox was localized mainly in the nuclei and partially in cytoplasm. We noted that some endometrial cancer cases with overexpressed β-catenin were also in concomitant with highly expressed Sox7. Interestingly, we observed that the highly expressed Sox7 was associated with the reduced expressions of CyclinD1 and FGF9 in these overexpressed β-catenin endometrial cancer samples, suggesting Sox7 has a negative regulatory role in suppressing Wnt/β-catenin signaling activity in endometrial cancer. In fact, using endometrial cancer and OEA cells lines as cell models further supported our notion that Sox7 could negatively regulate Wnt/β-catenin signaling activity and its tumorigenic capacities.

Mounting evidences have showed that aberrant activation of the Wnt/β-catenin signaling has associated with human cancers including endometrial cancer and ovarian endometrioid adenocarcinoma (OEA) [27-29]. Therefore, targeting Wnt/β-catenin signaling cascade is a potential good therapeutic approach to human cancers. The primary mediator of the oncogenic effects in this signaling pathway is β-catenin. Numerous studies have demonstrated that targeting the upstream effectors is able to inhibit Wnt/β-catenin signaling activity by reducing the level of β-catenin [30-33]. However, genetic mutation in β-catenin found in some human cancers such as endometrial cancer and OEA hinders the therapeutic approach of using inhibitors against upstream effectors in Wnt/β-catenin signaling cascade [34-36]. On the other hand, targeting the β-catenin/TCF protein complex will be a better choice in suppression of Wnt/β-catenin signaling activity. Indeed, numerous studies using small molecules have shown to inhibit this signaling activity successfully [37, 38]. Here we report Sox7 is a negative regulator of Wnt/β-catenin signaling and maybe a putative potential target in inhibition of this pathway activity. Our findings in this study provided several lines of evidence suggesting that Sox7 could suppress the transcriptional activity of β-catenin/TCF protein complex which harbors either wild-type or mutant β-catenin. According to our immunofluorescent microscopy, the nuclear co-localization indicated that there was a functional interaction among Sox7, β-catenin and TCF4. Indeed, further co-immunoprecipitation assay supported our notion of that there was a direct physical interaction between Sox7 with either β-catenin and TCF4. Based on these evidences, we hypothesized that the interaction of Sox7 might disrupt the transcriptional function of β-catenin/TCF/LEF-1 complex. However, further studies to investigate the interacting region and the binding effects of Sox7 and with β-catenin and TCF4 are warranted.

Although further studies are needed to delineate how the interaction of Sox7 mediated inhibition of -catenin/TCF/LEF-1 complex transcriptional activity, our findings collectively highlight the suppressive roles of Sox7 in Wnt/β-catenin signaling and its tumorigenic capacities in endometrial cancer cells carrying either wild-type or mutant β-catenin.

## Supplementary Figures


