# Supplementary material for: Down-regulation of Sox7 is associated with aberrant activation of Wnt/β-catenin signaling in endometrial cancer
Source: Oncotarget. 2012 Nov 7;3(12):1546–56. doi: 10.18632/oncotarget.667 (PMC3681493; doi:10.18632/oncotarget.667)
Supplement: Supplementary file 1 [file oncotarget-03-1546-s001.pdf]

Down-regulation of Sox7 is associated with aberrant activation of Wnt/β-catenin signaling in endometrial cancer - Chan et al

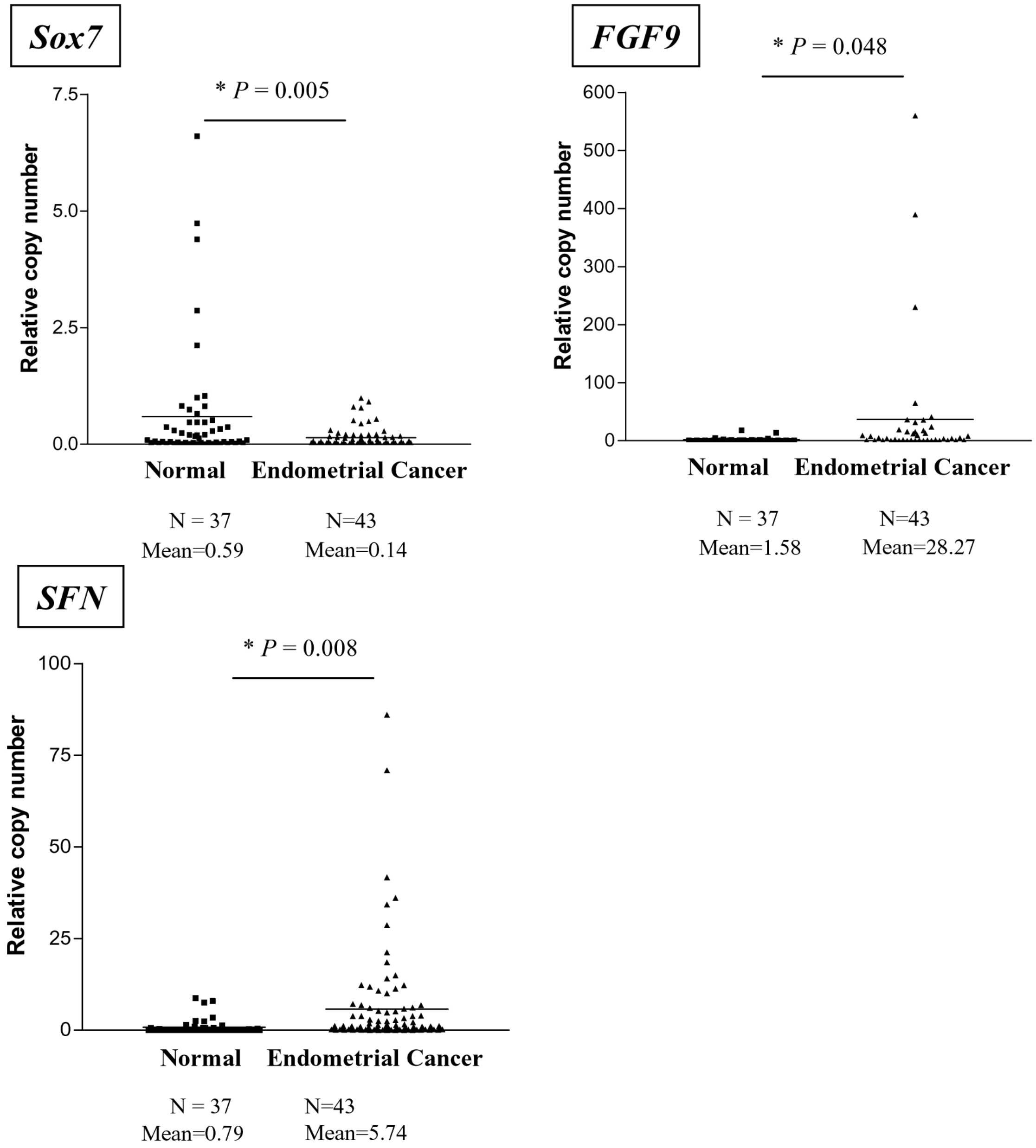

Supplementary Figure 1. Quantitative RT-PCR analysis showed that *Sox7* was significantly underexpressed in endometrial cancer ($P = 0.005$). Besides, the underexpressed was inversely correlated with two Wnt/β-catenin signaling targets; *FGF9* ($P = 0.0048$) and *SFN* ($P = 0.008$)
